# Exploring the relationship between serum magnesium levels, genetic variants and chronic kidney disease: a prospective study

**DOI:** 10.1093/ckj/sfaf254

**Published:** 2025-08-08

**Authors:** Sisi Xie, Idris Guessous, Dela Golshayan, Aurélien Thomas, Julien Vaucher, Pedro Marques-Vidal

**Affiliations:** Department of Medicine, Division of Medicine, Lausanne University Hospital and University of Lausanne, Lausanne, Switzerland; Primary care medicine service, Department of Primary Care Medicine, Geneva University Hospitals, Geneva, Switzerland; Department of Medicine, Transplantation Center, Lausanne University Hospital and University of Lausanne, Lausanne, Switzerland; Faculty Unit of Toxicology, CURML, Faculty of Biology and Medicine, University of Lausanne, Lausanne, Switzerland; Unit of Forensic Toxicology and Chemistry, CURML, Lausanne and Geneva University Hospitals, Lausanne, Geneva, Switzerland; Department of Medicine, Division of Medicine, Lausanne University Hospital and University of Lausanne, Lausanne, Switzerland; Department of Internal Medicine and Specialties, Division of Internal Medicine, Fribourg Hospital and University of Fribourg, Fribourg, Switzerland; Department of Medicine, Division of Medicine, Lausanne University Hospital and University of Lausanne, Lausanne, Switzerland

**Keywords:** chronic kidney disease, hypomagnesemia, mediation analysis, Mendelian randomization, single-nucleotide polymorphism

## Abstract

**Background:**

Recent evidence suggests that magnesium deficiency may play a role in the development and progression of chronic kidney disease (CKD). We assessed the association between genetic variations, serum magnesium levels and CKD risk in the general population.

**Methods:**

In this population-based prospective study (*n* = 4047; mean age 53 years; 54% female; mean follow-up 12.5 years), Cox regression models evaluated the effects of hypomagnesemia on CKD risk. Genetic risk scores and mediation analyses were used to assess the direct and indirect effects of single-nucleotide polymorphisms (SNPs) on CKD development through magnesium levels. One-sample and two-sample Mendelian randomization (MR) analyses were conducted to examine the causal relationship between genetically predicted serum magnesium levels and CKD risk.

**Results:**

Multivariable Cox regression analyses identified that hypomagnesemia was significantly associated with an increased risk of CKD, with a hazard ratio of 1.73 (95% confidence interval 1.14–2.61, *P* = .010). Analyses based on single SNPs and SNP scores did not reveal direct effects on CKD risk. Mediation analyses demonstrated that four SNPs exerted significant indirect effects on CKD risk through serum magnesium levels. However, the proportion of the total effect mediated by magnesium was low and not statistically significant. MR analyses did not provide strong evidence for a significant causal relationship between genetically predicted magnesium levels and CKD risk.

**Conclusions:**

Hypomagnesemia was significantly associated with an increased risk of long-term CKD. Genetic analysis suggests that serum magnesium levels may play an indirect role in CKD risk, but no clear causal relationship was found.

## Graphical Abstract

**Graphical Abstract:**
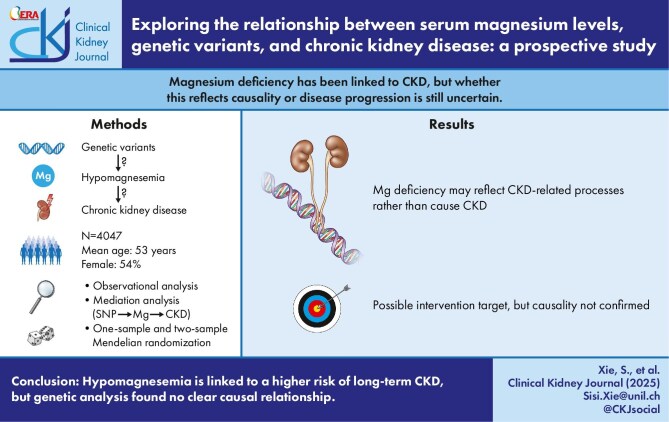

KEY LEARNING POINTS **What was known:**

- Hypomagnesemia is associated with chronic kidney disease (CKD) risk, but causality is unclear.
- Vitamin D deficiency is common in CKD, though its independent role remains inconsistent.
- The genetic link between serum magnesium and CKD risk was previously unclear.

**This study adds:**

- Baseline hypomagnesemia was independently associated with the long-term risk of CKD in a general population cohort.
- Coexisting hypomagnesemia and vitamin D deficiency significantly increased CKD risk.
- Mendelian randomization did not support a causal role for genetically predicted magnesium levels in CKD risk.

**Potential impact:**

- Hypomagnesemia may indicate early CKD rather than cause it; combined with vitamin D, it may aid in risk stratification and prevention.

## INTRODUCTION

Magnesium is an essential micronutrient and the second most abundant intracellular cation, playing a critical role in enzyme activation, cellular signaling, energy metabolism and numerous physiological functions, including cardiovascular and renal function [1]. Although magnesium metabolism is not directly regulated by traditional hormones, its balance primarily depends on intestinal absorption and renal excretion [2].

Chronic kidney disease (CKD) is a progressive condition characterized by the gradual loss of kidney function, which can ultimately progress to end-stage kidney disease (ESKD), requiring dialysis or kidney transplantation. Globally, more than 850 million individuals are affected by kidney diseases [3], making CKD a significant public health challenge. Studies suggest that adequate magnesium levels may help prevent various cardiovascular diseases and slow CKD progression, while low magnesium levels (hypomagnesemia) might be deleterious [4]. However, the association between hypomagnesemia and CKD is complex. On one hand, hypomagnesemia is highly prevalent among CKD patients, particularly in the early stages of the disease [5]. On the other hand, magnesium deficiency may accelerate CKD progression and is associated with poorer outcomes [6–9].

In addition to metabolic factors, genetic susceptibility plays a crucial role in the development and progression of CKD [10]. Single-nucleotide polymorphisms (SNPs), the most common type of genetic variation, can influence disease risk by regulating metabolic and physiological pathways [11]. In particular, SNPs associated with serum magnesium levels may reflect an individual's genetic susceptibility to CKD by modulating magnesium homeostasis [12]. However, the specific mechanisms by which genetic susceptibility through magnesium-related pathways may affect CKD progression remain incompletely understood.

Magnesium and vitamin D also share a close association with mineral metabolism and physiological function. Magnesium is critical for the synthesis, transport, and activation of vitamin D [13]. Although vitamin D was not a primary focus of this study, its role was considered as a potential confounder. This prospective population-based study aimed to assess the association between serum magnesium and long-term CKD risk, and to explore the potential contribution of genetic susceptibility through magnesium-related pathways.

## MATERIALS AND METHODS

### Population and study design

We used data from CoLaus|PsyCoLaus (www.colaus-psycolaus.ch), a population-based longitudinal study initiated in 2003 with 6733 middle-aged participants [14]. Baseline data were collected between 2003 and 2006, with three follow-ups conducted between 2009 and 2021. At inclusion, blood samples were collected from all participants, processed and stored for future analyses at –80°C.

### Genetic variants used in this study

Genome-wide genotyping was performed using the Affymetrix 500K SNP array. Participants of European ancestry were included based on grandparental origin. Standard quality control procedures were applied, including removal of related individuals and low-quality genotypes, and SNP filtering by minor allele frequency, call rate and Hardy–Weinberg equilibrium. Phasing and imputation were conducted using SHAPEIT2 and Minimac3 with the HRC reference panel.

SNPs significantly associated with serum magnesium levels were selected from the genome-wide association study (GWAS) catalog. Of the seven top variants initially selected, four SNPs—s4072037 (*MUC1*, mucin 1, cell surface associated), rs13146355 (*SHROOM3*, shroom family member 3), rs11144134 (*TRPM6*, transient receptor potential cation channel subfamily M member 6) and rs3925584 (*DCDC5*, doublecortin domain containing 5)—were available across all relevant datasets and were consistently used in downstream analyses to ensure methodological consistency. Among the four SNPs included, rs3925584 has been mapped to multiple nearby genes in different annotation sources, including *DCDC5, DCDC1* (*doublecortin domain containing 1*) and *MPPED2-AS1* (*MPPED2 antisense RNA 1*). For consistency and clarity, we report *DCDC5* as the representative gene throughout the manuscript. Detailed procedures are provided in the Supplementary data.

### Genetic instrument selection for MR

For the two-sample Mendelian randomization (MR) analysis, magnesium-associated SNPs were selected from a previously published GWAS by the CHARGE Consortium [15], which identified six independent genome-wide significant variants (*P* < 5 × 10⁻⁸) associated with serum magnesium in 15 366 European individuals. Of these, five SNPs (rs4072037, rs13146355, rs11144134, rs3925584 and rs7197653) were available in the CKDGen Consortium GWAS summary statistics on CKD risk (*N* > 1.2 million) [12]. During the harmonization process, one palindromic SNP (rs7197653) with intermediate allele frequency was excluded to avoid potential strand ambiguity. The final instrument, therefore, included four SNPs (rs4072037, rs13146355, rs11144134 and rs3925584) that were consistently present across both datasets. All SNPs were conditionally independent and not in linkage disequilibrium. Details of SNP characteristics, instrument strength and power estimates are provided in Supplementary data, Tables S1 and S2.

To validate the findings from the two-sample MR, we conducted a one-sample MR analysis using data from our study. This analysis also utilized the same four overlapping SNPs (rs4072037, rs13146355, rs11144134 and rs3925584), together with measured serum magnesium levels and CKD status, without reliance on external GWAS summary statistics. Details of SNP characteristics, instrument strength, and power estimates are provided in Supplementary data, Tables S3 and S4. All SNPs were conditionally independent and not in linkage disequilibrium.

### Measurement of kidney function, serum magnesium and 25 (OH) vitamin D

Serum creatinine levels were measured using the Jaffe method to estimate glomerular filtration rate (eGFR). eGFR was calculated according to the Chronic Kidney Disease Epidemiology Collaboration 2021 equation [16]. CKD was defined as an eGFR <60 mL/min/1.73 m^2^ body surface area [17]. Serum magnesium levels were measured using the xylidyl blue colorimetric method on a Cobas 8000 system (Roche Diagnostics, Switzerland). The within-run and total coefficients of variation for serum magnesium were 2.5% and 3.8%, respectively. Hypomagnesemia was defined by serum levels <1.8 mg/dL (0.74 mmol/L). Serum 25(OH)D₃ and 3-epi-25(OH)D₃ were quantified using a validated ultra-HPLC tandem-MS method. Vitamin D deficiency was defined as vitamin D concentrations <20 ng/mL, and vitamin D insufficiency as concentrations between 20 and <30 ng/mL [18].

### Relevant covariates

We included age, sex, education, marital status, weekly alcohol consumption, smoking status, hypertension, diabetes, body mass index (BMI) categories, high-sensitivity C-reactive protein (hs-CRP) and diuretic use, as well as the use of proton pump inhibitors (PPIs) and renin–angiotensin–aldosterone system (RAAS) inhibitors, as covariates. Further details on measurement procedures and definitions are provided in the Supplementary data.

### Exclusion criteria

For this study, we excluded participants with missing data on (i) follow-ups, (ii) eGFR, (iii) covariates, (iv) vitamin D and magnesium, and (v) SNPs.

### Ethical statement

The institutional Ethics Committee of the University of Lausanne, which afterwards became the Ethics Commission of Canton Vaud (www.cer-vd.ch), approved the CoLaus|PsyCoLaus study (project number PB_2018–00 038, reference 239/09). All participants gave their signed informed consent before entering the study.

### Statistical analysis

Statistical analyses were conducted using Stata v.18 (Stata Corp, College Station, TX, USA). Descriptive results were expressed as a number of participants (percentage) for categorical variables and as average ± standard deviation or median (interquartile range) for continuous variables. Between-group comparisons were conducted using chi-square for categorical variables and Student's *t*-test or Kruskal–Wallis test for continuous variables.

First, multivariable linear regression models were used to evaluate the association between SNPs and serum magnesium levels. Second, Cox proportional hazards regression models were used to assess the independent effects of baseline hypomagnesemia, vitamin D status and their interactions on CKD risk, with hazard ratios (HRs) and 95% confidence intervals (CIs) reported. Additionally, single SNP analyses and SNP score analyses were conducted to evaluate the direct effects of genetic variations on CKD risk. The SNP score was constructed by summing the number of magnesium-lowering alleles across the four selected SNPs (range: 0–7) and analyzed using Cox models to examine its association with CKD risk. To explore whether serum magnesium levels mediated the association between SNPs and CKD risk, bootstrap-based mediation analysis was performed to estimate direct effects, indirect effects and the proportion of mediation, using bootstrap iterations to compute confidence intervals for mediation effects.

Both one-sample and two-sample MR analyses were conducted using the TwoSampleMR package in RStudio (version 2024.12.0 + 467) to estimate the causal effect of genetically predicted serum magnesium levels on CKD risk. Statistical power for the primary analyses was calculated based on the variance explained (R²). For the two-sample MR, harmonization was performed to ensure consistent effect allele alignment across exposure and outcome datasets. Causal estimates were derived using the inverse-variance weighted (IVW) method. Sensitivity analyses included the weighted median and MR-Egger regression. Horizontal pleiotropy was assessed using the MR-Egger intercept (*P* > .05 indicating no evidence of pleiotropy), and heterogeneity was evaluated using Cochran's Q test. For the one-sample MR, SNP–magnesium and SNP–CKD associations were estimated using multivariable linear and logistic regression models, respectively, adjusting for predefined covariates including age, sex and the first three genetic principal components to account for population stratification.

Statistical significance was considered for a two-sided test with *P* < .05.

## RESULTS

### Selection of participants

**Figure 1:**
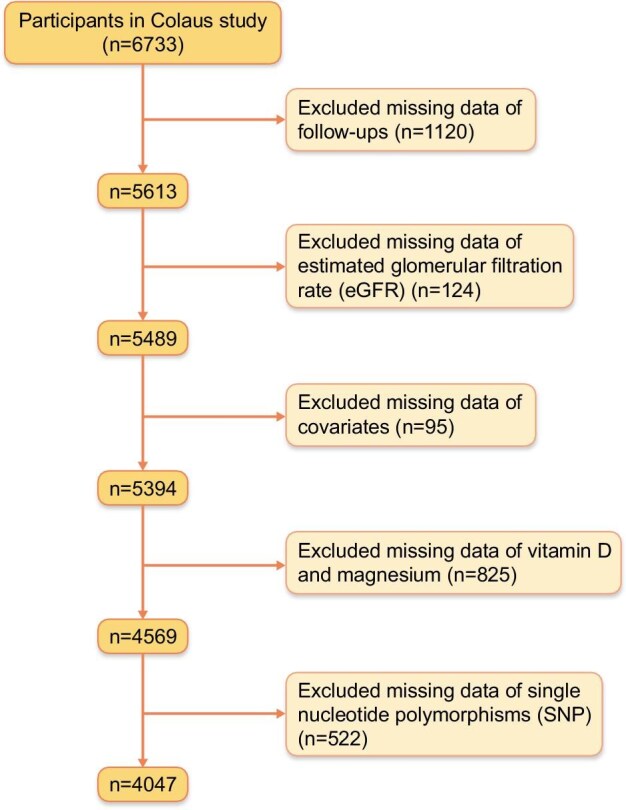
Flowchart of participants’ inclusion and exclusion.

We excluded participants with missing data on (i) follow-ups (*n* = 1120), (ii) eGFR (*n* = 124), (iii) covariates (*n* = 95), (iv) vitamin D and magnesium (*n* = 825), and (v) SNPs (*n* = 522). Finally, 4047 participants were included in this study (Fig. 1). The characteristics of included and excluded participants are presented in Supplementary data, Table S5. Excluded participants were younger, less likely to be female, and more likely to have a lower education level, be current smokers, abstain from alcohol and never engage in physical activity. They also had a higher prevalence of obesity, hypertension and diabetes, exhibited higher eGFR and hs-CRP levels, and had lower vitamin D levels compared with the included participants.

Among the participants selected for the study (mean age 53 years, 54% female), 3% presented with CKD at baseline. The mean eGFR was 88.6 mL/min/1.73 m^2^. Among participants, 4.60% presented with hypomagnesemia. The characteristics of the included participants according to the eGFR category are indicated in Table 1. Compared with the normomagnesemia group, participants in the hypomagnesemia group were more likely to have a lower education level, a higher prevalence of obesity, hypertension and diabetes, elevated uric acid levels, and significantly higher hs-CRP concentrations (Table 1).

**Table 1:** Baseline characteristics of participants by magnesium status.

Variables	Normal Mg level (≥1.8 mg/dL) (*n* = 3861)	Low Mg level (<1.8 mg/dL) (*n* = 186)	*P-*value
Age,years	52.9 ± 10.5	54.3 ± 10.6	.077
Female sex, *n* (%)	2083 (54.0)	102 (54.8)	.812
Education level, *n* (%)			**.003**
High	818 (21.2)	24 (12.9)	
Middle	938 (24.3)	38 (20.4)	
Low	2105 (54.5)	124 (66.7)	
Marital status, *n* (%)			.321
Living alone	1256 (32.5)	67 (36.0)	
Living in couple	2605 (67.5)	119 (64.0)	
Smoking status, *n* (%)			.145
Never	1583 (41.0)	71 (38.2)	
Former	1295 (33.5)	75 (40.3)	
Current	983 (25.5)	40 (21.5)	
Alcohol consumption, *n* (%)			.290
None	1001 (25.9)	51 (27.4)	
1–13/week	2212 (57.3)	96 (51.6)	
14–27/week	516 (13.4)	29 (15.6)	
28+/week	132 (3.4)	10 (5.4)	
BMI group, *n* (%)			**<.001**
Normal	1909 (49.4)	69 (37.1)	
Overweight	1423 (36.9)	69 (37.1)	
Obese	529 (13.7)	48 (25.8)	
Hypertension, *n* (%)	1338 (34.7)	89 (47.9)	**<.001**
Diabetes, *n* (%)	194 (5.0)	47 (25.3)	**<.001**
Physical activity, *n* (%)			.122
Never	1233 (31.9)	73 (39.3)	
Once a week	361 (9.4)	14 (7.5)	
Twice a week	2217 (57.4)	95 (51.1)	
Does not know	50 (1.3)	4 (2.1)	
Serum creatinine, µmol/L	80.2 ± 17.8	78.2 ± 17.6	.143
Uric acid, mmol/L	310.5 ± 83.0	326.6 ± 106.3	**.011**
hs-CRP, mg/L	1.2 (0.6–1.6)	2.0 (0.8–4.4])	**<.001**
Kidney function (%)			.123
No CKD	3750 (97.1)	177 (95.2)	
CKD	111 (2.9)	9 (4.8)	
eGFR, mL/min/1.73 m^2^	88.5 ± 14.8	89.8 ± 16.7	.237
Diuretics, *n* (%)	76 (2.0)	24 (12.9)	**<.001**
PPIs, *n* (%)	210 (5.4)	18 (9.7)	**.014**
RAAS inhibitors, *n* (%)	453 (11.7)	44 (23.7)	**<.001**
Vitamin D, ng/mL	21.1 ± 9.6	20.2 ± 9.9	.232
Vitamin D status, *n* (%)			
Deficiency	1930 (50.0)	98 (52.7)	
Insufficiency	1255 (32.5)	60 (32.3)	
Sufficient	676 (17.5)	28 (15.0)	.645

Results are expressed as the number of participants (percentage) for categorical variables and as average ± standard deviation or median (interquartile range) for continuous variables.

Between-group comparisons were performed using chi-square for categorical variables and Student's *t*-test or Kruskal–Wallis test for continuous variables.

Mg, magnesium.

Bold indicates statistically significant values (P < .05).

### Genetic variants and hypomagnesemia

The associations between single SNPs and hypomagnesemia are presented in Table 2. Significant associations were observed for rs4072037, rs3925584 and rs11144134, which were linked to an increased likelihood of hypomagnesemia (*P* < .05). In contrast, rs13146355 showed no significant association with hypomagnesemia. After applying Bonferroni correction for multiple comparisons, the statistical significance of the results remained unchanged.

**Table 2:** Association between SNPs and hypomagnesemia.

SNP	Unadjusted coefficient (95% CI)	*P-*value	Adjusted coefficient (95% CI)	*P-*value
rs4072037 (*MUC1*)	0.02 (0.02–0.03)	**<.001**	0.02 (0.02–0.03)	**<.001**
rs3925584 (*DCDC5*)	0.01 (0.01–0.02)	**.001**	0.01 (0.01–0.02)	**<.001**
rs11144134 (*TRPM6*)	0.02 (0.01–0.03)	**.006**	0.02 (0.01–0.03)	**.003**
rs13146355 (*SHROOM3*)	0.01 (0.00–0.02)	**.015**	0.01 (0.00–0.01)	.167

Multivariate model adjusted by sex, age, education level, marital status, smoking, alcohol consumption, diabetes, hypertension, BMI, physical activity, diuretics, CRP, baseline eGFR level, PPIs and RAAS inhibitors.

SNP identifiers are followed by mapped gene names in parentheses.

Bold indicates statistically significant values (P < .05).

### SNPs, SNP scores and long-term risk of CKD

The associations between individual SNPs, SNP scores and long-term risk of CKD are detailed in Table 3. Neither individual SNPs nor the SNP score showed significant associations with CKD risk. Furthermore, interaction analyses revealed no significant interactions between the SNP score and hypomagnesemia, or between the SNP score and vitamin D status (deficiency or insufficiency).

**Table 3:** Association between SNPs, SNP score and CKD risk.

Abbreviations	Unadjusted HR (95% CI)	*P*-value	Adjusted HR (95% CI)	*P*-value
Single SNP analysis				
rs4072037 (*MUC1*)	1.02 (0.88–1.20)	.771	1.02 (0.87–1.19)	.843
rs3925584 (*DCDC5*)	0.95 (0.82–1.11)	.533	0.88 (0.76–1.03)	.103
rs11144134 (*TRPM6*)	0.80 (0.58–1.09)	.157	0.88 (0.65–1.21)	.444
rs13146355 (*SHROOM3*)	1.12 (0.96–1.30)	.145	1.09 (0.93–1.28)	.264
SNP score analysis				
SNPscore	1.01 (0.93–1.10)	.838	0.98 (0.9–1.07)	.673
Interaction analysis				
SNPscore × hypomagnesemia	1.01 (0.74–1.38)	.943	1.06 (0.76–1.47)	.739
SNPscore × vitamin D deficiency	0.91 (0.72–1.16)	.444	1.00 (0.78–1.28)	.999
SNPscore × vitamin D insufficient	0.87 (0.68–1.12)	.274	1.04 (0.81–1.34)	.751

Multivariate model adjusted by sex, age, education level, marital status, smoking, alcohol consumption, diabetes, hypertension, BMI, physical activity, diuretics, CRP, baseline eGFR level, PPIs and RAAS inhibitors.

SNP identifiers are followed by mapped gene names in parentheses.

### Associations between baseline magnesium, vitamin D and long-term CKD risk

Among the 4047 participants initially considered, 120 had CKD at baseline and were excluded from the analysis. The remaining 3927 participants without CKD at baseline were followed for a median of 12.5 years (interquartile range: 4.9–16.9 years). During this period, 329 participants (8.4%) developed incident CKD, defined as an eGFR <60 mL/min/1.73 m² at follow-up.

As shown in Table 4, baseline hypomagnesemia was significantly associated with an increased risk of long-term CKD (multivariable-adjusted HR 1.73, 95% CI 1.14–2.61, *P* = .010). In contrast, baseline vitamin D status (deficiency or insufficiency) was not independently associated with the risk of CKD. However, when hypomagnesemia and vitamin D deficiency coexisted, the risk of long-term CKD increased significantly (multivariable-adjusted HR 2.03, 95% CI 1.22–3.38, *P* = .006).

**Table 4:** Association between baseline vitamin D, magnesium and CKD risk.

Abbreviations	Unadjusted HR (95% CI)	*P*-value	Adjusted HR (95% CI)	*P*-value
Hypomagnesemia	1.97 (1.33–2.90)	**.001**	1.73 (1.14–2.61)	**.010**
Vitamin D status				
Vitamin D deficiency	1.00 (0.73–1.36)	.986	1.24 (0.90–1.70)	.187
Vitamin D insufficient	1.09 (0.79–1.50)	.609	1.30 (0.94–1.80)	.119
Both deficiency	2.84 (1.77–4.58)	**<.001**	2.03 (1.22–3.38)	**.006**

Multivariate model adjusted by sex, age, education level, marital status, smoking, alcohol consumption, diabetes, hypertension, BMI, physical activity, diuretics, CRP, baseline eGFR level, PPIs and RAAS inhibitors.

Bold indicates statistically significant values (P < .05).

### Mediation analysis

The mediation analysis results, summarized in Table 5, indicate that SNPs rs4072037, rs3925584 and rs11144134 exerted significant indirect effects on the long-term risk of CKD through magnesium levels (all *P* < .05). However, the proportion of the total effect mediated by magnesium was not statistically significant.

**Table 5:** Bootstrap results for mediation analysis.

SNP	Indirect effect coefficient (95% CI)	Direct effect coefficient (95% CI)	Total effect coefficient (95% CI)	Proportion of indirect to total effect coefficient (95% CI)	*P-*value of indirect effect
rs4072037 (*MUC1*)	−0.03 (−0.06 to −0.01)	0.03 (−0.18 to 0.23)	−0.004 (−0.21 to 0.20)	6.70 (−99.81 to 113.22)	**.008**
rs3925584 (*DCDC5*)	−0.02 (−0.03 to −0.002)	−0.14 (−0.33 to 0.05)	−0.16 (−0.35 to 0.04)	0.11 (−1.51 to 1.73)	**.030**
rs11144134 (*TRPM6*)	−0.03 (−0.05 to −0.001)	−0.38 (−0.79 to 0.02)	−0.41 (−0.82 to −0.002)	0.07 (−0.59 to 0.72)	**.045**
rs13146355 (*SHROOM3*)	−0.01 (−0.02 to 0.005)	0.14 (−0.05 to 0.32)	0.13 (−0.05 to 0.32)	−0.05 (−2.06 to 1.96)	.238

Multivariate model adjusted by sex, age, education level, marital status, smoking, alcohol consumption, diabetes, hypertension, BMI, physical activity, diuretics, CRP, baseline eGFR level, PPIs and RAAS inhibitors.

SNP identifiers are followed by mapped gene names in parentheses.

Bold indicates statistically significant values (P < .05).

### MR analysis

#### Two-sample MR analysis

The primary two-sample MR analysis was conducted using four SNPs that were consistently available in both the CHARGE (exposure) and CKDGen (outcome) datasets. Using the IVW method, there was no evidence of a causal association between genetically predicted serum magnesium levels and CKD risk (β = –0.35, 95% CI –1.03 to 0.32, *P* = .300; Table 6). Similarly, the weighted median method yielded a non-significant estimate (β = 0.02, 95% CI –0.03 to 0.08, *P* = .434), and MR-Egger regression produced a positive association (β = 1.47, 95% CI 0.96 to 1.98, *P* = .030). However, the MR-Egger intercept suggested potential directional pleiotropy (intercept = –0.015, *P* = .018), and Cochran's Q test revealed substantial heterogeneity (Q = 744.94, *P* = 3.78 × 10⁻¹⁶).

**Table 6:** Two-sample MR analysis of the association between genetically predicted serum magnesium levels and CKD risk.

Analysis	Estimate (95% CI)	*P*-value	Heterogeneity (Q, *P*)	Horizontal pleiotropy (intercept, *P*)
Primary analysis				
IVW	–0.35 (–1.03, 0.32)	.300	744.94 (*P* = 3.78e-161)	
Sensitivity analyses				
Weighted median	0.02 (−0.03, 0.07)	.434		
MR-Egger	1.47 (0.96, 1.98)	**.030**	26.69 (*P* = 1.60e-06)	−0.015 (*P* = .018)

Estimates are log ORs per 1-SD increase in genetically predicted serum magnesium.

Heterogeneity assessed by Cochran's Q; pleiotropy by MR-Egger intercept.

Bold indicates statistically significant values (P < .05).

The mean F-statistic of the instruments was 24.77 (Supplementary data, Table S2). Power calculations indicated that the study had 99.99% power to detect an effect size of odds ratio (OR) = 1.2. Leave-one-out analysis indicated that the causal estimate was particularly sensitive to the exclusion of rs4072037, which contributed disproportionately to the overall effect (Supplementary data, Fig. S1).

#### One-sample MR analysis

The primary one-sample MR analysis based on four SNPs available in the CoLaus dataset showed no significant association between genetically predicted serum magnesium levels and CKD risk using the IVW method (β = –0.96, 95% CI –6.24 to 4.32, *P* = .722; Table 7). Consistent findings were obtained using the weighted median method (β = –0.99, 95% CI –7.41 to 5.43, *P* = .763) and MR-Egger regression (β = –2.07, 95% CI –14.91 to 10.78, *P* = .753). The MR-Egger intercept suggested no evidence of horizontal pleiotropy (intercept = 0.04, *P* = .853), and Cochran's Q test indicated minimal heterogeneity across instruments (Q = 0.09, *P* = .993).

**Table 7:** One-sample MR analysis of the association between genetically predicted serum magnesium levels and CKD risk.

Analysis	Estimate (95% CI)	*P*-value	Heterogeneity (Q, *P*)	Horizontal pleiotropy (intercept, *P*)
Primary analysis				
IVW	−0.96 (−6.24, 4.32)	.722	0.09 (*P* = .993)	
Sensitivity analyses				
Weighted median	−0.99 (−7.41, 5.43)	.763		
MR-Egger	−2.07 (−14.91, 10.78)	.753	0.06 (*P* = .971)	0.04 (*P* = .853)

Estimates are log odds ratios per 1-SD increase in genetically predicted serum magnesium.

Heterogeneity assessed by Cochran's Q; pleiotropy by MR-Egger intercept.

The mean F-statistic of the instruments was 5.96. However, power calculations (R² = 0.586%) indicated 90.1% power to detect an OR of 1.2 (Supplementary data, Table S4, Fig. S2 presents the leave-one-out analysis from the one-sample MR. The causal estimates remained stable across iterations, indicating that no individual SNP exerted a disproportionate influence on the overall association.

## DISCUSSION

### Main findings

This study systematically examined associations between serum magnesium, vitamin D levels, genetic factors and long-term CKD risk. Baseline hypomagnesemia was significantly associated with increased CKD risk, whereas vitamin D status alone was not. Coexisting hypomagnesemia and vitamin D deficiency further elevated the risk, suggesting synergy. Mediation analysis showed several SNPs had indirect effects via magnesium, though the proportion mediated was not statistically significant. One- and two-sample MR analyses found no causal relationship between genetically predicted magnesium levels and CKD risk.

### Comparison with previous studies

Our findings on hypomagnesemia and CKD risk are consistent with previous observational studies. Several cohorts, including diabetic populations and large-scale CKD registries, have reported that low serum magnesium levels predict kidney function decline and adverse outcomes [6, 7, 19–21]. A recent meta-analysis also linked hypomagnesemia to increased cardiovascular and all-cause mortality in CKD and ESKD patients [9]. However, not all studies agree; for instance, Azem *et al.* did not find an association between hypomagnesemia and the risk of eGFR decline [8]. This discrepancy may be attributed to differences in study design, population characteristics and statistical approaches.

Our study adds to this body of evidence by using an MR approach, which differs fundamentally from observational studies. By leveraging genetic instruments, we aimed to infer causality while minimizing residual confounding and reverse causation. While our results did not support a strong causal effect, this does not necessarily contradict prior epidemiological findings. Instead, it suggests that previously reported associations may be driven by confounding factors or that the causal effect, if present, is modest and requires larger sample sizes for detection.

### Mechanistic insights

The association between hypomagnesemia and CKD risk likely reflects magnesium's role in renal homeostasis. Low magnesium levels have been linked to oxidative stress, inflammation, mitochondrial dysfunction and dysregulated calcium signaling, contributing to vascular constriction and hypertension [6, 22–25]. Magnesium also appears to mitigate renal injury by inhibiting tubular phosphate-calcium crystallization [26]. By suppressing vascular smooth muscle cell calcifications, magnesium also reduces coronary and peripheral artery disease-related events in CKD patients [27–29]. Furthermore, magnesium may reduce cardiovascular mortality and morbidity in CKD patients through parathyroid hormone inhibition [30].

From a genetic perspective, mediation analysis suggests that certain SNPs may influence CKD risk indirectly via magnesium levels. TRPM6 encodes a magnesium transporter, and its mutations may impair magnesium absorption in the gut and reabsorption in the kidney, leading to hypomagnesemia [31, 32]. MUC1, which encodes transmembrane mucin involved in the intestinal barrier, has a regulatory SNP (e.g. rs4072037) associated with serum magnesium levels, possibly affecting paracellular magnesium absorption [33]. The roles of *SHROOM3* and *DCDC5* in magnesium regulation are not fully known; both genes are strongly associated with eGFR and serum creatinine, suggesting a potential influence on renal tubular magnesium handling [15]. However, the proportion of indirect effects to total effects was not statistically significant. This finding highlights two possible scenarios: (i) SNPs primarily act through magnesium with negligible direct effects, or (ii) multiple opposing pathways suppress the total effect.

To further examine the causal role of serum magnesium in CKD, we conducted two-sample MR analyses using SNPs identified from a large GWAS. The primary IVW method did not reveal a significant association between genetically predicted serum magnesium levels and CKD risk. While the MR-Egger regression suggested a positive effect, it was accompanied by evidence of directional pleiotropy. Sensitivity analyses, including the weighted median method, supported the null association. Consistent findings were observed in the one-sample MR analysis using individual-level data from the CoLaus cohort. Collectively, these results suggest that magnesium likely reflects CKD-related processes rather than causing disease.

In addition to the mechanistic links discussed above, the association between hypomagnesemia and CKD may also reflect underlying metabolic and inflammatory disturbances rather than a direct causal pathway. In our study, hypomagnesemia was strongly associated with multiple known CKD risk factors, including obesity, hypertension, diabetes, hyperuricemia and systemic inflammation (Table 1). These conditions are not only well-established contributors to CKD development but may also influence serum magnesium levels through various mechanisms. For example, diabetes can increase urinary magnesium excretion due to osmotic diuresis and impaired tubular reabsorption [34]; chronic inflammation may lead to altered magnesium transport and redistribution through oxidative stress pathways [35]; and commonly prescribed antihypertensive diuretics can promote kidney magnesium loss [36]. These shared pathways suggest that hypomagnesemia may function more as a surrogate marker of high-risk metabolic states than as an independent driver of CKD.

### Strengths and limitations

This study has several strengths. First, it combined observational, mediation, and both one-sample and two-sample MR analyses to comprehensively assess the interplay between serum magnesium, genetic factors and CKD risk. Second, by quantifying the indirect effects of SNPs on CKD via magnesium, we provided insights into magnesium's potential mediatory role and clinical relevance. However, this study also has limitations. First, potential weak instrument bias in one-sample MR (F-statistic = 5.96). Second, statistical power was limited by the modest number of CKD cases (*N* = 329). Third, residual confounding from unmeasured variables cannot be excluded. Fourth, all participants in this study were of European ancestry, which may limit the generalizability of our findings to other ethnic populations. Finally, the CoLaus cohort represents a relatively low-risk population for incident CKD, as evidenced by a baseline CKD prevalence of 3.0% and a 12.5-year incident CKD rate of 8.4%. This may limit the generalizability of our findings to higher-risk populations, such as those with diabetes or albuminuria. Moreover, data on albuminuria, such as urine albumin-to-creatinine ratio, a key marker of kidney damage, were unavailable, which restricted our ability to stratify baseline CKD risk more precisely.

## CONCLUSION

Hypomagnesemia is associated with CKD risk, but MR analysis does not support a causal relationship. Magnesium may act as a biomarker of CKD progression rather than a causal factor.

## Supplementary Material

sfaf254_Supplemental_File

## Data Availability

The data of CoLaus|PsyCoLaus study used in this article cannot be fully shared as they contain potentially sensitive personal information on participants. According to the Ethics Committee for Research of the Canton of Vaud, sharing these data would be a violation of the Swiss legislation with respect to privacy protection. However, coded individual-level data that do not allow researchers to identify participants are available upon request to researchers who meet the criteria for data sharing of the CoLaus|PsyCoLaus Datacenter (CHUV, Lausanne, Switzerland). Any researcher affiliated to a public or private research institution who complies with the CoLaus|PsyCoLaus standards can submit a research application to research.colaus@chuv.ch or research.psycolaus@chuv.ch. Proposals requiring baseline data only, will be evaluated by the baseline (local) Scientific Committee (SC) of the CoLaus and PsyCoLaus studies. Proposals requiring follow-up data will be evaluated by the follow-up (multicentric) SC of the CoLaus|PsyCoLaus cohort study. Detailed instructions for gaining access to the CoLaus|PsyCoLaus data used in this study are available at www.colaus-psycolaus.ch/professionals/how-to-collaborate/.
