# Supplementary material for: Exploring the relationship between serum magnesium levels, genetic variants and chronic kidney disease: a prospective study
Source: Clin Kidney J. 2025 Aug 8;18(9):sfaf254. doi: 10.1093/ckj/sfaf254 (PMC13014121; doi:10.1093/ckj/sfaf254)
Supplement: sfaf254_Supplemental_File [file sfaf254_supplemental_file.docx]

**Supplementary information**

**Supplementary methods**

We selected age (years), sex (male/female), education, marital status, weekly alcohol consumption, smoking, hypertension (yes/no), diabetes (yes/no), body mass index (BMI) categories, high-sensitivity c-reactive protein (hs-CRP), and diuretics(yes/no) were included.

Education was categorized into high (university), middle (high school) and low (apprenticeship + mandatory). Marital status was defined as living alone (single, divorced, widowed) or living with a partner. Usual alcohol consumption during the week was self-reported and reported as number of units (glasses of wine, bottles or cans of beer, and shots of spirits) per week. Smoking was self-reported and categorized as never, former (irrespective of the time since quitting smoking) and current.

Body weight and height were measured with participants barefoot and in light indoor clothes. Body weight was measured in kilograms to the nearest 100 g using a Seca® scale (Hamburg, Germany). Height was measured to the nearest 5 mm using a Seca® (Hamburg, Germany) height gauge. BMI was calculated and categorized as normal (<25 kg/m^2^), overweight ≥25 and <30 kg/m^2^) and obese ≥30 kg/m^2^). Blood pressure (BP) was measured using an Omron® HEM-907 automated oscillometric sphygmomanometer after at least a 10-minute rest in a seated position, and the average of the last two measurements was used. Hypertension was defined by a SBP ≥140 mm Hg or a DBP ≥90 mm Hg or the presence of antihypertensive drug treatment. Glucose was assessed by glucose dehydrogenase. Diabetes mellitus was defined as fasting plasma glucose ≥7.0 mmol/L and/or the presence of oral hypoglycaemic or insulin treatment.

Participants reported all medicines prescribed or bought over the counter. Medicines were coded according to the Anatomic, Therapeutic and Chemical classification of the WHO. Diuretics were defined by code C03*, Proton pump inhibitors (PPIs) were defined by code A02*, and RAAS inhibitors (including ACE inhibitors and ARBs) were defined by codes C09*, respectively. where *=any code.

## **Genetic variants used in this study**

Genome-wide genotyping was performed using the Affymetrix 500K SNP array. Nuclear DNA was extracted from the whole blood of participants considered of Caucasian origin, defined as having all four grandparents born in a selected number of countries. Genotypes were called using BRLMM (https://tools.thermofisher.com/content/sfs/brochures/500K_data_analysis_workflow_brlmm.pdf). Duplicate individuals, and first- and second-degree relatives, were identified and removed by computing estimates of pairwise genomic kinship coefficients, using KING[1]. Subjects were excluded from the analysis in case of inconsistency between sex and genetic data, a genotype call rate <90%, or inconsistencies of genotyping results in duplicate samples. SNPs were filtered using the following quality control criteria: monomorphic (or with minor allele frequency (MAF) <1%), call rates <90%, and deviation from the Hardy-Weinberg equilibrium (HWE) (p <1x10-06). Phased haplotypes were generated using SHAPEIT2[2, 3]. Imputation was performed using minimac3 and the Haplotype Reference Consortium (HRC version r1.1)[4] hosted on the Michigan Imputation Server[5].

SNPs related to magnesium levels were selected from an online database (https://www.ebi.ac.uk/gwas/efotraits/EFO_0004845, assessed 17.10.2024). Among the seven SNPs most significantly associated with magnesium levels, only five (rs4072037, rs13146355, rs11144134, rs3925584, and rs4460629) were available in the dataset provided to us, due to non-availability in the genotyping data.

**References**

1. Manichaikul A, Mychaleckyj JC, Rich SS*, et al.* Robust relationship inference in genome-wide association studies. Bioinformatics 2010;26(22):2867-2873

2. Delaneau O, Marchini J, Zagury JF. A linear complexity phasing method for thousands of genomes. Nat Methods 2011;9(2):179-181

3. Delaneau O, Zagury JF, Marchini J. Improved whole-chromosome phasing for disease and population genetic studies. Nat Methods 2013;10(1):5-6

4. McCarthy S, Das S, Kretzschmar W*, et al.* A reference panel of 64,976 haplotypes for genotype imputation. Nat Genet 2016;48(10):1279-1283

5. Das S, Forer L, Schönherr S*, et al.* Next-generation genotype imputation service and methods. Nat Genet 2016;48(10):1284-1287

**Supplementary Table 1**: Characteristics of SNPs used as genetic instruments for serum magnesium in two-sample MR.

| **SNP** | **Mapped genes** | **Chr.** | **Position** | **Risk allele** | **Other allele** | **Beta** | **SE** | ***P*-value** | **EAF** |
| --- | --- | --- | --- | --- | --- | --- | --- | --- | --- |
| rs4072037 | MUC1 | 1 | 155162067 | C | T | -0.01 | 0.001 | 2.01E-36 | 0.46 |
| rs13146355 | SHROOM3 | 4 | 77412140 | G | A | -0.005 | 0.001 | 6.27E-13 | 0.56 |
| rs11144134 | TRPM6 | 9 | 77499796 | T | C | -0.011 | 0.001 | 8.21E-15 | 0.92 |
| rs3925584 | DCDC5 | 11 | 30760335 | C | T | -0.006 | 0.001 | 5.20E-16 | 0.45 |

SNP: single nucleotide polymorphism; Chr: chromosome; SE: Standard Error; *MUC1*: mucin 1, cell surface associated; *SHROOM3*: shroom family member 3; *TRPM6:* transient receptor potential cation channel subfamily M member 6; *DCDC5*: doublecortin domain containing 5~~;~~ ~~PRMT7:~~*~~Protein Arginine Methyltransferase 7~~.*

**Supplementary Table 2**: Instrument strength and statistical power calculations in two-sample MR.

| **Metric** | **Value (primary analysis)** |
| --- | --- |
| Number of SNPs | 4 |
| Mean F-statistic | 24.77 |
| Variance explained (R²) | 2.39% |
| Power (OR= 1.2) | 99.9998% |

**Supplementary Table 3**: Characteristics of SNPs used as genetic instruments for serum magnesium in one-sample MR.

| **SNP** | **Mapped genes** | **Chr.** | **Position** | **Risk allele** | **Beta** | **SE** | ***P*-value** | **EAF** |
| --- | --- | --- | --- | --- | --- | --- | --- | --- |
| rs4072037 | *MUC1* | 1 | 155,162,067 | C | -0.005 | 0.015 | 0.767 | 0.46 |
| rs13146355 | *SHROOM3* | 4 | 77,412,140 | G | 0.026 | 0.006 | 4.83e-06 | 0.56 |
| rs11144134 | *TRPM6* | 9 | 77,499,796 | T | 0.055 | 0.012 | 4.84e-06 | 0.92 |
| rs3925584 | *DCDC1*; *MPPED2-AS1* | 11 | 30,760,335 | C | -0.051 | 0.011 | 4.83e-06 | 0.45 |

SNP: single nucleotide polymorphism; Chr: chromosome; SE: Standard Error; *MUC1*: mucin 1, cell surface associated; *HMGN2P18*: high mobility group nucleosomal binding domain 2 pseudogene 18; *KRTCAP2*: keratinocyte associated protein 2; *SHROOM3*: shroom family member 3; *TRPM6:* transient receptor potential cation channel subfamily M member 6; *DCDC1*: doublecortin domain containing 1; *MPPED2-AS1*: MPPED2 antisense RNA 1

**Supplementary Table 4**: Instrument strength and statistical power calculations in one-sample MR.

| **Metric** | **Value (primary analysis)** |
| --- | --- |
| Number of SNPs | 4 |
| Mean F-statistic | 5.96 |
| Variance explained (R²) | 0.586% |
| Power (OR= 1.2) | 90.1% |

**Supplementary Table 5** Baseline characteristics of included and excluded participants.

| **Variables** | **Included**  **(n=4047 )** | **Excluded (n=2686)** | ***P-value*** |
| --- | --- | --- | --- |
| Age,years | 53.0 ± 10.5 | 52.1 ± 11 | **0.002** |
| Female sex, % | 2185 (54.0) | 1359 (50.6) | **0.006** |
| Education level, % |  |  | **0.010** |
| High | 842 (20.8) | 478 (17.9) |  |
| Middle | 976 (24.1) | 649 (24.3) |  |
| Low | 2229 (55.1) | 1545 (57.8) |  |
| Marital status, % |  |  | 0.628 |
| Living alone | 1323 (32.7) | 891 (33.3) |  |
| Living in couple | 2724 (67.3) | 1788 (66.7) |  |
| Smoking status, % |  |  | **<0.001** |
| Never | 1654 (40.9) | 1078 (40.2) |  |
| Former | 1370 (33.8) | 813 (30.3) |  |
| Current | 1023 (25.3) | 789 (29.5) |  |
| Alcohol consumption (%) |  |  | **<0.001** |
| None | 1052 (26.0) | 863 (32.1) |  |
| 1-13/week | 2308 (57.0) | 1345 (50.1) |  |
| 14-27/week | 545 (13.5) | 368 (13.7) |  |
| 28+/week | 142 (3.5) | 110 (4.1) |  |
| BMI group, % |  |  | **<0.001** |
| Normal | 1978 (48.9) | 1259 (46.9) |  |
| Overweight | 1492 (36.9) | 970 (36.2) |  |
| Obese | 577 (14.2) | 454 (16.9) |  |
| Hypertension, % | 1427 (35.3) | 1073 (40.1) | **<0.001** |
| Diabetes, % | 241 (6.0) | 195 (7.3) | **0.028** |
| Physical activity |  |  | **<0.001** |
| Never | 1306 (32.3) | 1071 (41.3) |  |
| Once a week | 375 (9.3) | 276 (10.7) |  |
| Twice a week | 2312 (57.1) | 1195 (46.1) |  |
| Does not know | 54 (1.3) | 50 (1.9) |  |
| Serum creatinine, umol/L | 80.1 ± 17.8 | 79.3 ± 25.5 | 0.122 |
| Uric acid, mmol/l | 311.2 ± 84.3 | 314 ± 85.5 | 0.186 |
| High-sensitivity CRP, mg/L | 1.2 [0.6-2.6] | 1.4 [0.7-2.9] | **<0.001** |
| Kidney function (%) |  |  | 0.990 |
| Normal | 3927 (97) | 2590 (97) |  |
| CKD | 120 (3) | 79 (3) |  |
| eGFR, mL/min/1.73m^2^ | 88.6 ± 14.9 | 91.1 ± 15.7 | **<0.001** |
| Vitamin D, ng/ml | 21.1 ± 9.6 | 18.7 ± 9.5 | **<0.001** |
| Magnesium,mg/dl | 2.1 ± 0.2 | 2.1 ± 0.2 | 0.105 |

BMI, body mass index; CRP, C-reactive protein; CKD, chronic kidney disease. Results are expressed as number of participants (percentage) for categorical variables and as average ± standard deviation or median [interquartile range] for continuous variables. Between-group comparisons performed using chi-square for categorical variables and student’s t-test or Kruskal-Wallis test for continuous variables.

**Supplementary Figure 1.** Leave-one-out analysis for the two-sample MR of serum magnesium on CKD.

**Supplementary Figure 2.** Leave-one-out analysis for the one-sample MR of serum magnesium on CKD.
